# Caffeine Synthesis and Its Mechanism and Application by Microbial Degradation, A Review

**DOI:** 10.3390/foods12142721

**Published:** 2023-07-17

**Authors:** Zhipeng Lin, Jian Wei, Yongqiang Hu, Dujuan Pi, Mingguo Jiang, Tao Lang

**Affiliations:** 1 School of Chemistry and Chemical Engineering, Guangxi Minzu University, Nanning 530008, China; l2888977@163.com; 2 Guangxi Key Laboratory for Polysaccharide Materials and Modifications, School of Marine Sciences and Biotechnology, Guangxi Minzu University, Nanning 530008, China; huyongqiang2129@163.com (Y.H.); dujuanpi@163.com (D.P.); 3 Institute of Ecology, College of Urban and Environmental Sciences and Key Laboratory for Earth Surface Processes of Ministry of Education, Peking University, Beijing 100091, China; weijianpku@stu.pku.edu.cn; 4 MNR Key Laboratory for Geo-Environmental Monitoring of Great Bay Area & Shenzhen Key Laboratory of Marine Bioresource and Eco-Environmental Science, College of Life Sciences and Oceanography, Shenzhen University, Shenzhen 518071, China

**Keywords:** caffeine, N-demethylation, C-8 oxidation, methylxanthine, microorganisms

## Abstract

Caffeine is a metabolite derived from purine nucleotides, typically accounting for 2–5% of the dry weight of tea and 1–2% of the dry weight of coffee. In the tea and coffee plants, the main synthesis pathway of caffeine is a four-step sequence consisting of three methylation reactions and one nucleosidase reaction using xanthine as a precursor. In bacteria, caffeine degradation occurs mainly through the pathways of N-demethylation and C-8 oxidation. However, a study fully and systematically summarizing the metabolism and application of caffeine in microorganisms has not been established elsewhere. In the present study, we provide a review of the biosynthesis, microbial degradation, gene expression, and application of caffeine microbial degradation. The present review aims to further elaborate the mechanism of caffeine metabolism by microorganisms and explore the development prospects in this field.

## 1. Introduction

Caffeine (1,3,7-trimethylxanthine) is a xanthine alkaloid that is derived from plants. It was first isolated from coffee seeds by German scientists Runge and Von Giese in 1820 [1,2]. Owing to its multifaceted biological effects, caffeine has been found widespread use in everyday life, as well as in the food and medical industries.

In the food industry, caffeine is used as a natural additive in various products, such as chocolate, tea, and cola. Not only does it significantly improve the taste of food [3], but it also can alleviate fatigue and improve sustained attention [4]. Numerous studies have shown that moderate caffeine consumption can obviously promote diuresis [5], improve memory, increase anticancer effects [6,7,8,9,10], decrease blood pressure [11], lower the risks of type 2 diabetes [12,13], and delay aging [14]. In the medical field, caffeine is primarily used as a stimulant [15] and an analgesic adjuvant [16]. Moreover, caffeine has been identified as a potential biomarker for disease detection, especially in the case of Parkinson’s disease [17,18,19]. Apart from these applications, the cosmetics industry has also developed caffeine-based products for weight loss and beauty care. Caffeine can promote skin blood microcirculation, prevent fat accumulation, and provide antioxidant benefits [20].

However, the use of caffeine also poses severe environmental challenges. For example, the production of coffee and tea generates millions of tons of agricultural and industrial refuse annually. As a result, the scientific community and the public are highly concerned about the environmental impact from caffeine and its biodegradation. Although there have been some reports on the metabolic pathways of caffeine in microorganisms, a comprehensive summary is currently lacking. In this review, we mainly focus on the metabolic pathways of caffeine degradation in bacteria and fungi, and we summarize the structures and gene sequences of related enzymes. Our aim is to further elucidate the mechanism of caffeine metabolism in microorganisms, and provide theoretical references for further fundamental research, utilization, and development of caffeine in the future.

## 2. Caffeine Biosynthesis in Plants and Its Ecological Functions

Caffeine was first detected in coffee and tea plants, and it has been identified in more than 100 plant species. In particular, some *Camellia* and *Coffea* species possess relatively high levels of caffeine. For example, *Coffea arabica* and *Camellia sinensis* contain 1–2% and 2–5% caffeine, respectively. Other plants, such as *Theabronma cacao*, *Cola acuminata*, *Ilex paraguariensis*, and *Paullinia cupana*, contain 0.03%, 1.5%, 0.7%, and 4% caffeine, respectively [21].

### 2.1. Pathways for Caffeine Synthesis in Plants

Currently, caffeine synthesis is primarily derived from tea and coffee plants, in which its pathway of synthesis is largely similar. This pathway consists of a core pathway and a xanthosine synthesis pathway, both of which contribute to the formation of caffeine [21].

#### 2.1.1. Caffeine Biosynthesis Pathways from Xanthosine in Plants

**Figure 1:**
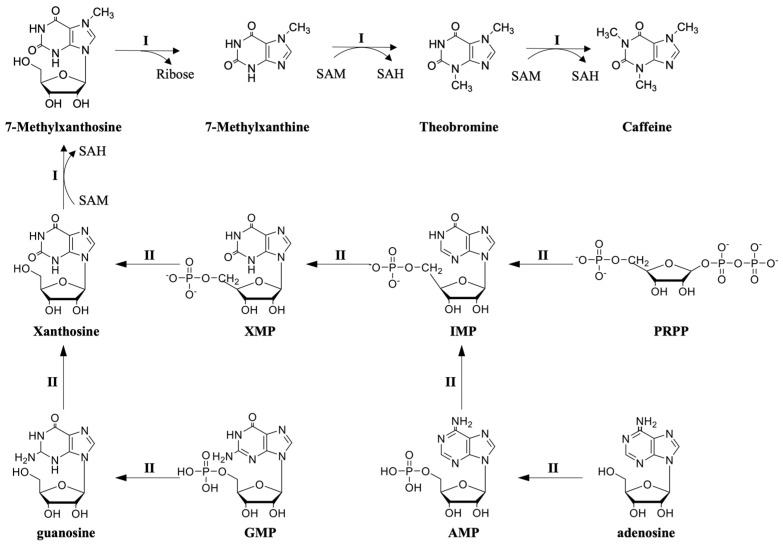
The biosynthesis pathway of caffeine in tea plants. The core pathways for caffeine synthesis in tea plants are shown as I. The synthesis pathways of xanthosine in tea plants are shown as II. Arrows represent the synthetic routes. Note: PRPP, 5-phosphoribose-1-pyrophosphate; IMP, 5′-monophosphate; AMP, adenosine monophosphate; XMP, xanthosine-5′monophosphate; GMP, guanosine 5′-Monophosphate; SAH, S-adenosyl-_L_-homocysteine; and SAM, S-adenosyl-_L_-methionine.

The core pathway of caffeine synthesis can be divided into four steps, which involve three methylation reactions catalyzed by three different types of N-methyltransferases and one nucleosidase reaction catalyzed by N-methylnucleosidase [22]. The specific pathway is xanthosine → 7-methylxanthosine → 7-methylxanthine → theobromine → caffeine (route I in Figure 1), where xanthosine serves as the substrate [23]. The purine ring of caffeine primarily comes from purine nucleotides, and the methyl donor involved in the methylation process is S-adenosyl-L-methionine (SAM) [24].

#### 2.1.2. Pathways Supplying Xanthosine for Caffeine Biosynthesis in Plants

Xanthosine serves as the initiator of caffeine synthesis and can be synthesized through four main pathways (route II in Figure 1):

1. The de novo route. The de novo route is the most significant synthesis pathway of xanthosine in caffeine production. This pathway starts from 5-phosphoribose-1-pyrophosphate (PRPP), which is transformed into inosine 5′-monophosphate (IMP) via a series of enzyme-catalyzed reactions and ultimately converted into xanthosine (the key enzymes involved in the conversion from IMP to xanthosine are IMP dehydrogenase and 5′-nucleotidase) [21].
2. The AMP route. Xanthine nucleosides can be synthesized from adenine nucleosides in the following way: AMP → IMP → XMP → xanthosine (the key enzymes involved in the conversion from AMP to xanthosine are AMP deaminase, IMP dehydrogenase, and 5′-nucleotidase) [25].
3. The SAM cycle route. The synthesis of xanthosine can also occur through S-adenosylhomocysteine (SAH) in the S-adenosylmethionine (SAM) cycle [26]. The pathway from SAH to xanthine nucleosides involves the following steps: SAH → adenosine → adenine → AMP → IMP → XMP → xanthosine (the key enzymes involved in the conversion from adenosine to xanthosine are adenosine nucleosidase, adenine phosphoribosyltransferase, AMP deaminase, IMP dehydrogenase, and 5′-nucleotidase).
4. The GMP route. Guanine nucleotides can also be involved in the synthesis of caffeine [27,28], which is synthesized in the following pathway: GMP → guanosine → xanthosine (the key enzyme involved in the conversion from GMP to xanthosine is guanosine deaminase).

### 2.2. Ecological Function of Caffeine

There are two main ecological functions of caffeine: allelopathy (inhibition of the growth of other plants) and chemical defense (protection against pathogens and animals).

Allelopathy refers to the effects of caffeine released by plants on neighboring plant seeds and seedlings [29]. However, mature plant litter can also release caffeine into the surrounding environment, eventually affecting the environment. A previous study showed that mature coffee trees produce about 150–200 g (dry weight)/m^2^/year of litter, which releases about 1–2 g caffeine/m^2^/year into the soil [30]. However, the extent to which caffeine is involved in allelopathy is still unclear since soil contains microorganisms that can metabolize a great deal of caffeine, and some caffeine may be also degraded by soil bacteria. Moreover, the antibacterial activity of caffeine may reduce the catabolism of caffeine in soil, prolonging its retention time and increasing its accumulation. In addition, high concentrations of caffeine (>5 mM) can have toxic effects on the plant itself and affect seed germination. When the concentration of caffeine exceeds 10 mM, it can completely inhibit the growth of radicles [31].

Chemical defense refers to the defensive effects of caffeine contained in the young leaves, fruits, and flower buds of plants such as tea and coffee [32]. It can prevent the young and soft tissues from damage caused by microbial pathogens and herbivores [32]. Interestingly, after the gene for caffeine synthesis was transferred into tobacco in vitro, it was observed that the transgenic tobacco had a significant improvement in resistance to pests and diseases [33]. In addition, Hollingsworth et al. (2008) found that low concentrations (1–2%) of caffeine can be used as an insect repellent, effectively killing snails and slugs without damaging plants [34]. More interestingly, caffeine can also kill harmful pathogenic microorganisms by promoting the growth of their natural enemies [35]. This method of cultivating the enemy of the harmful organisms is a very powerful weapon for plant development.

## 3. Caffeine Metabolism in Bacteria

Worldwide, at least 71 bacterial strains belonging to 27 genera are involved in caffeine degradation [36], among which *Pseudomonas* is the most common and studied caffeine-degrading strain. In *Pseudomonas*, the metabolism of caffeine is composed of two metabolic pathways: N-terminal demethylation and C-terminal oxidation. These two degradation pathways often coexist in bacteria, and the N-demethylation pathway is the main pattern of caffeine degradation in bacteria.

### 3.1. N-Demethylation Pathway

N-demethylation is the most common mechanism of caffeine degradation in bacteria, and it is also the main caffeine degradation pathway in bacteria, such as *Pseudomonas* [37], *Serratia* [38], etc. At present, *Pseudomonas putida* CBB5 is the most studied bacterium in the N-demethylation pathway, and it is used as a representative organism to explain the mechanism of bacterial N-demethylation degradation of caffeine in the present paper.

**Figure 2:**
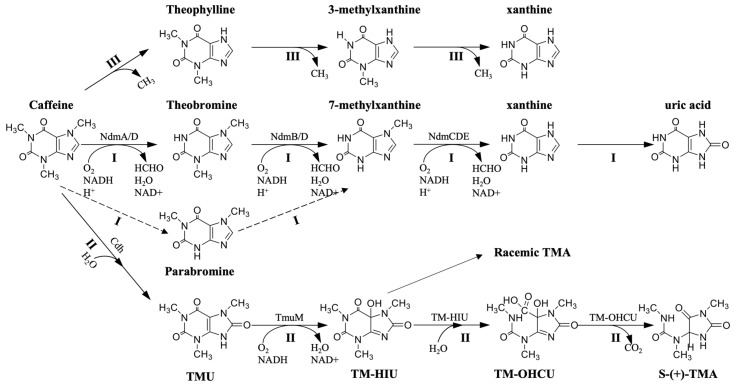
The known degradation pathway of caffeine in microorganisms. The pathway of N-demethylation in *Pseudomonas* CBB1 is shown as I. The pathway of C-8 Oxidative in *Pseudomonas* CBB1 is shown as II. The pathway of caffeine in fungi is shown as III. Solid arrows represent major pathways. Dashed arrows represent secondary pathways.

In *Pseudomonas putida* CBB5, caffeine is transported into the bacteria [39], and then multiple N-demethylases [40,41,42] break down the three N-methyl bonds to form xanthine. The enzymes involved in caffeine catabolism are known to be inducible. During the demethylation, caffeine binds to the C-terminus of NdmA (N-demethylase A) in a hexamer composed of NdmA and NdmB. NdmA catalyzes the demethylation of caffeine N-1 methyl to theobromine (major product) and paraxanthine (minor product) [43]. Theobromine subsequently binds to the C-terminal active site of NdmB in the hexamer, which catalyzes the N-demethylation of the N-3 methyl group of theobromine to 7-methylxanthine. In both pathways, NdmA and NdmB are coupled to NdmD, which transfers electrons from NADH to NdmA with NdmB, and ultimately, 7-methylxanthine is N-demethylated to xanthine by the NdmCDE protein complex (route I in Figure 2). Throughout the process, one molecule each of NADH and O_2_ are consumed to remove one methyl group and generate one formaldehyde [44].

After caffeine is demethylated to xanthine, it is oxidized to uric acid by xanthine oxidase. Uric acid is then further oxidized to allantoin by urate oxidase, which is subsequently hydrolyzed to allantoic acid by allantoinase. Allantoic acid is further hydrolyzed to urea and glyoxylic acid. Finally, urea is decomposed into CO_2_ and NH_3_ by the action of urease [45].

### 3.2. C-8 Oxidation Pathway

The C-8 oxidative degradation pathway of caffeine has been widely found in *Klebsiella*, *Rhodococcus* [46,47], *Pseudomonas* [48], and *Alcaligenes* [49]. Among them, the C-8 oxidation pathway of *Pseudomonas* sp. CBB1 has been studied in the most detail, and this section mainly uses it as a representative to elaborate the C-8 oxidative metabolism pathway of caffeine degradation (route II in Figure 2).

In 1998, Madyastha and Sridhar discovered the first step of the oxidation reaction of caffeine C-8, in which caffeine was hydrolyzed to 1.3.7-trimethyluric acid (TMU) catalyzed by caffeine dehydrogenase (Cdh) [46]. Subsequently, in 2012, Mohanty et al. found the second step of the C-8 oxidation pathway: TMU is hydroxylated to 1,3,7-trimethyl-5-hydroxyisourate (TM-HIU), which is catalyzed by an NADH-dependent trimethyl-uric acid monooxygenase (TmuM); in the third reaction, TM-HIU is spontaneously converted to racemic 3,6,8-trimethylallantoin (TMA) by the intermediate 3,6,8-trimethyl-2-oxo-4-hydroxy-4-carboxy-5-ureidoimidazolin (TM-OHCU) or generates TM-OHCU catalyzed by TM-HIU hydrolase and then generates S-(+)-TMA catalyzed by TM-OHCU decarboxylase [50].

## 4. Characteristics of Structures and Sequences from the Enzymes in the Bacterial Degradation Pathway of Caffeine

### 4.1. Structural Features of N-Demethylases

In vitro, N-demethylase is mainly in the supernatant after cleavage, but in the purification process, the N-demethylase activity will be quickly lost [51], which might lead to many difficulties for the analysis of the N-demethylation mechanism and structure of N-demethylase. At present, the structures and sequences of five demethylases (NdmABCDE) in *Pseudomonas putida* CBB5 are relatively clear. These five enzymes are all monomeric enzymes, among which NdmA and NdmB are Rieske monooxygenases containing nonheme iron active centers with molecular weights of 40 kDa and 35 kDa, respectively [52].

Recent biochemical and structural studies have shown that Rieske nonheme iron-dependent oxygenases (ROs) have an α3 or α3β3 configuration [53]. Like other oxidases, ROs have a catalytic core that binds one (monooxygenase) or two (dioxygenase) atoms of O_2_ to the target site using transition metals (iron, copper, and manganese) and cofactors (NAD(P)H, and flavin) [54]. In *Pseudomonas putida* CBB5, the [2Fe-2S] cluster in the N-terminal Rieske domain of the α subunit of the RO enzyme is anchored to the substrate through coordination with two histidine residues and two cysteine residues. The active-site iron atom, which is located in the C-terminal domain, is then coordinated with two histidine residues and one aspartic acid/glutamate residue.

NdmA and NdmB are highly similar in sequence and structure and possess N-1- and N-3-specific N-demethylation activities, respectively [55]. Both enzymes are composed of two domains: the N-terminal Rieske domain containing [2Fe-2S], which accepts foreign electrons, and the C-terminal SRPBCC domain containing a nonheme iron center, which binds ligands and oxidizes substrates (an image of a three-dimensional protein structure is referred to [55]). The Rieske domain of NdmA contains three α helices and nine β strands. The secondary structure of NdmB is similar to that of NdmA, but with a ring structure replacing the α3 helix. The [2Fe-2S] clusters of NdmA and NdmB are located between β4-α3 and β6-β7, respectively, and are coordinated with two cysteine and two histidine residues. The nonheme iron center forms an octahedral arrangement with two histidines, one aspartic acid, and water molecules on the catalytic face. NdmA is 44.7 Å away from the Rieske [2Fe-2S] cluster and nonheme iron within the NdmB monomer but is only 12.1 Å away from its neighboring subunits. This result suggests that electrons are transferred from the Rieske [2Fe-2S] cluster of the α subunit to the nonheme iron center of the neighboring α subunit. In contrast to the Rieske domain, the SRPBCC domain exhibits a relatively variable conformation, consistent with its role in substrate selectivity. During the demethylation process, NdmA and NdmB tend to form an α3–α′3 heterohexamer structure, which accelerates the demethylation reaction due to the larger interface area, leading to the more efficient degradation of caffeine.

In terms of the kinetics of enzyme-catalyzed reactions, NdmA and NdmB are both non-substrate-specific enzymes capable of degrading a variety of methylxanthines [56] and exhibit different catalytic efficiencies for different substrates (Table 1) [44]. NdmA catalyzes the N-1 demethylation of caffeine, theophylline, paraxanthine, and 1-methylxanthine, producing theobromine, 3-methylxanthine, 7-methylxanthine, and xanthine, respectively, with a catalytic efficiency in the order of theophylline > caffeine > paraxanthine > 1-methylxanthine. In contrast, NdmB catalyzes the N-3 demethylation of caffeine, theophylline, theobromine, and 3-methylxanthine, producing paraxanthine, 7-methylxanthine, 1-methylxanthine, and xanthine, respectively, with a catalytic efficiency in the order of theobromine > 3-methylxanthine > theophylline > caffeine. The difference in catalytic efficiency of different N-methyl groups is determined by the distance between the N-methyl group of the substrate methylxanthine and the nonheme iron in NdmA and NdmB [55]. When the SRPBCC domain of NdmA binds to the substrate caffeine and theophylline, its active center is closer to the N-1 methyl group than the other methyl groups, leading to the highest enzyme activity. The distance from the SRPBCC domain to the N-1 methyl group of theophylline is greater than that of caffeine; therefore, the catalysis efficiency of theophylline is higher than that of caffeine.

NdmC is another monooxygenase enzyme that binds substrates with a ligand-binding domain in its C-terminus [57]. Unlike NdmA and NdmB, NdmC lacks the core Rieske oxygenase domain [2Fe-2S] cluster in its N-terminus. In vitro, when co-expressed with NdmD and NdmE, NdmC forms a soluble multi-subunit protein complex called NdmCDE with a molecular weight of 360 kDa. NdmCDE consists of three NdmC, three NdmD, and three NdmE subunits (3α3β3γ) and is specific for the substrate 7-methylxanthine. However, when expressed alone, NdmC is expressed as inclusion bodies, indicating that it requires the presence of NdmD and NdmE for proper folding and solubility.

NdmD is a 65 kDa reductase belonging to the FNR reductase family, which couples NADH to NdmA and NdmB for electron transfer. The N-terminus of NdmD contains a conserved Rieske-type [2Fe-2S] domain, which is essential for electron transfer to NdmC. NdmD also contains a flavin group, an NADH-binding FR-type reductase domain, and a plant-type [2Fe-2S] domain that coordinates the iron–sulfur cluster to four cysteine residues [58]. The FR-type reductase domain is a type of flavin reductase domain. NdmD is partially soluble (<5%) when expressed in *E. coli* alone but is soluble when the NdmCDE complex was formed. NdmE, identified as a member of the GST superfamily, is a noncatalytic subunit that plays a structural role in the complexation of NdmC and NdmD [57], as well as a key role in the solubility and formation of the NdmCDE complex.

### 4.2. Gene Sequence Characteristics of N-Demethylases

Bacterial genes involved in caffeine degradation exhibit certain sequence characteristics. For example, *Pseudomonas* sp. S31, S32, S37, and S60 contain the complete NdmABCDE gene within the same gene cluster. However, some bacteria, such as *Acinetobacter* sp. S40, only have partial N-demethylase genes, and their Ndm genes are not colocalized. Nonetheless, Ndm genes are typically flanked by similar genes, such as ABC family and other transporters, permeases, GntR family, LysR family, and MarR family transcriptional regulators, regardless of the presence or absence of the complete NdmABCDE gene [36].

**Figure 3:**
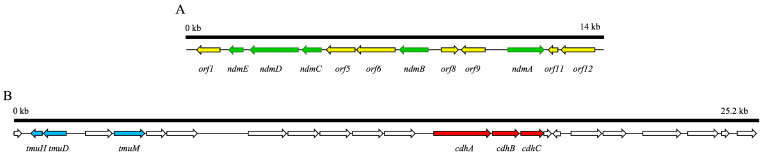
Characteristics of structures and sequences from the enzymes in the bacterial degradation pathway of caffeine. (**A**) Sequence arrangement of N-demethylase-related genes in the genome of *Pseudomonas* sp. NCIM 5235 [58]. Arrows suggest the position and orientation of each *orf*. Green arrows suggest the genes directly involved in caffeine metabolism, and yellow arrows indicate genes involved in the metabolism of by-products and other unknown functions. (**B**) Sequence arrangement of genes involved in the C-8 pathway in the genome of *Pseudomonas* sp. CBB1 [50]. Arrows suggest the position and orientation of each *orf*. Genes required for caffeine oxidation are represented by blue arrows, while those required for trimethyluric acid oxidation are represented by red arrows.

Currently, there are numerous studies investigating the gene sequences of *Pseudomonas putida* CBB5, *Pseudomonas* sp. NCIM 5235, and *Paraburkholderia caffeinilytica* CF1 strains, which have the ability to degrade caffeine. The gene of *Pseudomonas* sp. NCIM 5235 has been sequenced, and its NdmABCDE gene sequence and length are similar to those of *Pseudomonas putida* CBB5 [58]. The caffeine-metabolism-related gene is about 14 kb in size (Figure 3A) and contains the NdmABCDE enzyme gene for demethylation and other functional genes. *Orf*1 (open reading frame 1) and *orf*12 are involved in formaldehyde metabolism and the main by-product formed after the demethylation of methylxanthine. *Orf*6 encodes a membrane protein homologous to purine permease, which may act as a transporter for methylxanthine in the cell. In addition, *Orf*8 encodes a transcription regulator belonging to the GntR family, which likely acts as a transcriptional repressor of methylxanthine degradation. The protein encoded by *orf*11 belongs to the VOC family and may play a role in oxygen chelation in the cell, thus contributing to oxidation reactions. The functions of *orf*5 and *orf*9 in methylxanthine metabolism remain unknown (Table 2). In the *Paraburkholderia caffeinilytica* CF1 strain, complete functional demethylation genes are also present, but the difference is that its caffeine degradation gene is located on a giant plasmid of 174 kb [59]; this was the first time that caffeine-metabolism-related genes have been found to exist on a plasmid.

### 4.3. Structure and Gene Sequence Characteristics of C-8 Dehydrogenase

Currently, *Pseudomonas* sp. CBB1 is the most extensively studied strain among bacteria that degrade caffeine through the C-8 oxidative pathway. Two enzymes, Cdh and TmuM, involved in the caffeine C-8 oxidation pathway have been purified and characterized. Cdh, which acts as a heterotrimer, is composed of α, β, and γ subunits with molecular weights of 90, 32, and 20 kDa, respectively [48]. To be specific, the α subunit contains molybdopterin cofactor, the β subunit contains FAD cofactor, and the γ subunit contains iron–sulfur clusters. The optimal electron acceptor is co-enzyme Q0, which serves as the heterotrimeric caffeine dehydrogenase to oxidize caffeine at the C-8 position. The enzyme, which is specific for caffeine, possesses very low activity for theobromine and no activity for xanthine [50]. TmuM is a monooxygenase that belongs to the FAD family and contains the FAD-binding domain with NADH. Each TmuM molecule can bind one FAD, which consumes one molecule of O_2_ and one molecule of NADH to form one TMA molecule. TmuM was not active on uric acid but was active on methyluric acid, with the highest activity observed against trimethyluric acid (Table 3).

After sequencing the genome of *Pseudomonas* sp. CBB1, a 25.2 kb gene was identified, consisting of 21 complete ORFs [50]. A homology comparison revealed several genes involved in the C-8 pathway, including cdhA, cdhB, and cdhC, encoding α, β, and γ subunits, respectively. The tmuM gene encodes TmuM, while the tmuH and tmuD genes are highly similar to the hiu-hydrolase gene of *Rhizobium leguminosarum* and the HCu-decarboxylase gene of *Starkeya novella*, respectively, indicating their respective functions are encoding TM-HIU hydrolase and TM-OHCU decarboxylase (Table 4). The genomic data provide valuable insights into the molecular mechanisms of caffeine degradation by *Pseudomonas* sp. CBB1.

## 5. Caffeine Metabolism in Fungi

There have been relatively few studies on caffeine degradation by fungi. However, several fungi capable of degrading caffeine have been isolated and identified, including *Aspergillus*, *Rhizopus* [60], *Penicillium* [61], *Fusarium* [62], *Chrysosporium,* and *Gliocladium* [63]. When *Penicillium* and *Aspergillus* were cultured in vitro, the rate of caffeine degradation reached almost 100% [64]. Unlike the caffeine degradation pathway in bacteria, the primary degradation pathway in fungi involves 7-demethylation of caffeine to theophylline [65], followed by 1-demethylation of theophylline to 3-methylxanthine [66,67], and finally, degradation of 3-methylxanthine to xanthine (route III in Figure 2). The conversion of caffeine to theophylline is the rate-limiting step in caffeine metabolism by fungi. However, the specific molecular mechanism underlying caffeine degradation by fungi remains unclear.

## 6. Application of Caffeine Degradation by Microorganisms 

The daily average consumption of caffeine for humans ranges from 80 to 400 mg/person [68]. Excessive intake of caffeine can lead to various health problems, such as insomnia, anxiety [69], osteoporosis [70], and fetal weight loss [71]. Even low doses of caffeine can affect the quality of sleep [72,73] and have harmful effects on individuals with heart disease and on pregnant women. Caffeine is also considered an addictive substance [74], and its withdrawal can cause adverse reactions, such as headache, fatigue, apathy, and drowsiness [75,76,77]. Moreover, caffeine is recognized as an environmental pollutant [78,79,80], and the problem of how to remove it has garnered increasing attention from the scientific community.

Currently, traditional methods are used globally for caffeine removal, including water extraction, adsorption [81], supercritical CO_2_ extraction [82,83], microwave-assisted extraction [84,85], ultrasound-assisted extraction [86], and membrane filtration [87,88]. However, these methods often come with issues, e.g., high costs, toxicity, or a lack of specificity for caffeine. Instead, caffeine biodegradation provides a safe and inexpensive alternative to physical and chemical methods [89,90]. It mainly consists of two methods, that is, the microbial and enzymatic methods. The microbial method is more economical and eco-friendly in treating sewage and other waste containing caffeine and is preferred due to its safety and nontoxicity. Transgenic methods are being explored to produce strains that can remove caffeine, but this topic is relatively sensitive, and additionally, whether it can be utilized remains to be discussed. Enzymatic degradation of caffeine is mainly used in food applications and the production of specific intermediates, such as theophylline, 7-methylxanthine, and paraxanthine. Enzymatic degradation has the advantage of specifically removing methylxanthine, thus preserving the unique flavor and aroma compounds in coffee and tea, and avoiding an impact on the taste of food. However, caffeine-degrading enzymes are not stable in the environment [91], and their production and development costs are very high, leading to rare usage.

### 6.1. Reuse of Coffee Wastes

Coffee processing generates millions of tons of waste annually, which contain numerous high-value compounds, such as polyphenols, fibers, pigments, proteins, and polysaccharides [92]. Different types of this waste have enormous economic potential. Coffee peel or pulp can be utilized as feed for farm animals and can serve as a substrate for fermentation to prepare N-demethylase [93]. However, caffeine can decrease the palatability of and acceptance by animals to the fruit shell and pulp. Fortunately, microorganisms can quickly degrade caffeine and allow coffee waste to partially replace traditional animal feed additives [94]. Furthermore, coffee grounds can be used as a substrate for the cultivation of edible fungi. The cultivation of *Pleurotus ostreatus* [95,96] and *Flammulina velutipes* [97] by the application of coffee waste grounds has become a new method of its recycling.

### 6.2. Purifying Caffeine Wastewater

Caffeine, which is also widely present in aquatic environments, has been found to possess negative impacts on aquatic organisms, including oxidative stress, lipid peroxidation, neurotoxicity, energy reserve, and metabolic disorders, and meanwhile, it causes adverse effects on their reproduction, and development, and even causes mortality in some cases. Therefore, caffeine is now considered a new type of pollutant [98]. Although caffeine generally exhibits high removal efficiency in wastewater treatment processes, the increasing consumption of coffee and other caffeinated beverages has led to a higher input of caffeine into aquatic systems than its degradation amount [79]. Given the concerning levels of caffeine concentration in biotic and abiotic samples from related studies, it is important to develop suitable treatment methods and effective removal technologies to eliminate or significantly limit caffeine and its derivatives in aquatic systems.

Currently, *Pseudomonas* has been demonstrated to have good tolerance to caffeine and the ability to completely degrade caffeine in wastewater treatment [99,100]. Therefore, the application of *Pseudomonas* for caffeine degradation in sewage treatment will significantly improve the issue of damaged ecological environments and provide a new method for sewage purification.

### 6.3. Production of Methylxanthine

Methylxanthine is the main product of caffeine degradation and can offer various health benefits. It has anti-inflammatory and anti-oxidative properties, making it useful in the treatment of asthma [101]. It also has neuroprotective effects and can be used to treat neurodegenerative diseases [102]. Theophylline, a type of methylxanthine, possesses anti-inflammatory effects on chronic obstructive pulmonary disease and asthma [103,104]. Moreover, theobromine, another type of methylxanthine, has an obvious anticough effect without side effects [105], and paraxanthine can be used in the treatment of Parkinson’s disease [106]. 7-methylxanthine can also prevent the development of myopia and slow axial growth in children [107]. Compared to traditional synthesis methods, the biodegradation method of methylxanthine has significant advantages, such as being environmentally friendly, nontoxic, and economically beneficial. Currently, a variety of methylxanthines have been prepared and purified through biological fermentation, biocatalytic production, and purification of the high-value biochemical paraxanthine [108,109,110,111], which can provide a feasible approach for subsequent large-scale production.

**Figure 4:**
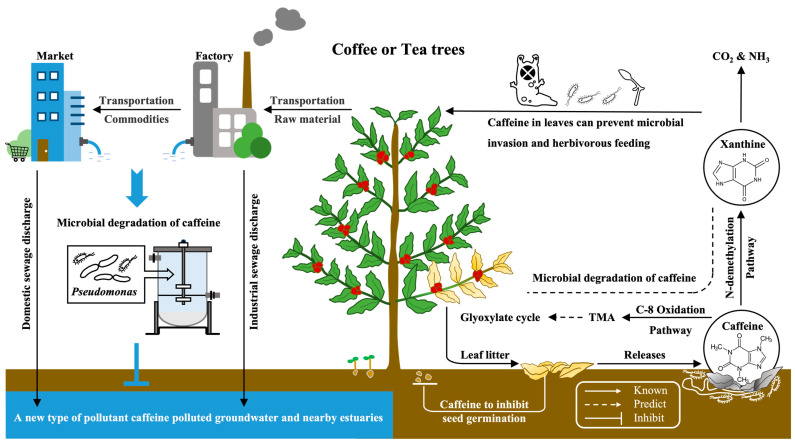
A schematic working model elaborating the caffeine biosynthesis in plants and degradation in microorganisms and its innovative application against caffeine pollutants.

Caffeine is a natural protector of plants against pests and microorganisms in their environment. As the leaves mature and fall, the caffeine is released into the soil, which inhibits the germination of seeds in the surrounding plants. Certain microorganisms, such as *Pseudomonas*, are able to break down caffeine through N-demethylation and C-8 oxidation pathways. However, in our daily lives, caffeine can enter the water circulation system through the processing of plant-based ingredients and food consumption. Excessive amounts of caffeine can lead to water pollution, but the microbial degradation of caffeine provides a new method for addressing this issue (Figure 4).

## 7. Conclusions

This study provides a comprehensive review of the natural synthesis and degradation pathways of caffeine. Plants are the main source of natural caffeine, and the synthesis pathway includes the core pathway of xanthine nucleoside to caffeine and the supplementary pathway of xanthine nucleoside synthesis. Bacterial degradation of caffeine follows two metabolic pathways, N-demethylation and C-8 oxidation, with N-demethylation being the primary degradation pathway. The N-demethylation pathway degrades caffeine to xanthine through N-demethylase (NdmABCDE). NdmABC is a monooxygenase, while NdmA and NdmB carry out the first two demethylation reactions, and NdmCDE complex is responsible for the last demethylation reaction. Complete N-demethylases are typically located in the same gene cluster, and their genes are usually flanked by specific genes. The C-8 oxidation pathway degrades caffeine to TMA by dehydrogens and oxygenases. Fungi, on the other hand, typically produce theophylline, 3-methylxanthine, and xanthine as the products of caffeine degradation.

Microbial degradation offers significant advantages over traditional caffeine removal methods in terms of reduced cost and the production of high-value methylxanthines. These findings indicate substantial commercial potential for caffeine biodegradation. While there are currently limited methods available for microbial caffeine degradation and the production of specific high-value by-products, further elucidation of the enzyme–substrate complex crystal structure is essential for optimizing these approaches. As software tools such as RoseTTAFold and AlphaFold2 continue to advance in accurately predicting protein spatial structures, the directed modification and de novo design of amino acid sequences for caffeine-degrading enzymes are expected to become feasible in the near future. Moreover, elucidating the mechanisms of fungal caffeine degradation may involve preliminary identification of potential fungal degrading enzyme gene sequences through sequence alignment and protein structure comparison. Additionally, the utilization of suitable fungal protein expression systems and the development of artificial cell membranes could play a critical role in unraveling the mechanisms underlying fungal caffeine degradation.

## Figures and Tables

**Table 1:** Kinetic parameters of the enzymatic reaction of NdmA and NdmB to methylxanthine [44].

Enzyme	Substrate	Product	*K_m_* (μM)	*k_cat_* (min^−1^)	*k_cat_*/*K_m_* (min^−1^·μΜ^−1^)
NdmA-His_6_	Caffeine	Theobromine	37 ± 8	190 ± 10	5.1 ± 1.2
	Theophylline	3-methylxanthine	9.1 ± 1.7	83 ± 1.7	9.1 ± 1.7
	Paraxanthine	7-methylxanthine	53 ± 20	130 ± 10	2.5 ± 0.8
	Theobromine	–	>500	NA	NA
	1-methylxanthine	Xanthine	270 ± 50	16 ± 1	0.06 ± 0.01
	3-methylxanthine	–	>500	NA	NA
	7-methylxanthine	–	>500	NA	NA
NdmB-His_6_	Caffeine	Paraxanthine	42 ± 9	0.23 ± 0.03	0.006 ± 0.001
	Theophylline	Methylxanthine	170 ± 50	0.27 ± 0.03	0.016 ± 0.005
	Paraxanthine	–	>500	NA	NA
	Theobromine	7-methylxanthine	25 ± 5	46 ± 1.9	1.8 ± 0.4
	1-methylxanthine	–	>500	NA	NA
	3-methylxanthine	Xanthine	22 ± 5	32 ± 1.5	1.4 ± 0.3
	7-methylxanthine	–	>500	NA	NA

**Table 2:** Proposed functions of genes present around RO reductase NdmD in gDNA of *Pseudomonas* sp. [58].

Gene	Homologous Protein	% Identity	Organism/NCBI Accession Number	Proposed Function
*orf1*	S-formylglutathione hydrolase	75	*Pseudomonas fluorescens* WP_017340027.1	Involved in formaldehyde metabolism
*NdmE*	Glutathione S-transferase	86	*Pseudomonas fluorescens* WP_017340028.1	Structural protein/chaperone
*NdmD*	Oxidoreductase	81	*Pseudomonas fluorescens* WP_080995163.1	Reductase component of methylxanthine demethylases
*NdmC*	Aromatic-ring-hydroxylating dioxygenase subunit alpha	88	*Pseudomonas fluorescens* WP_017340030.1	Methyxanthine N7-demethylation
*orf5*	Hypothetical protein	92	*Pseudomonas fluorescens* WP_017340031.1	Unknown
*orf6*	Purine permease	88	*Pseudomonas fluorescens* WP_017340032.1	Transport of methylxanthines
*NdmB*	Aromatic-ring-hydroxylating dioxygenase subunit alpha	91	*Pseudomonas fluorescens* WP_017340033.1	Methyxanthine N3-demethylation
*orf8*	GntR family transcriptional regulator	91	*Pseudomonas fluorescens* WP_017340034.1	Repressor protein of methylxanthine operon
*orf9*	Membrane protein	83	*Pseudomonas fluorescens* WP_017340035.1	Unknown
*NdmA*	Aromatic-ring-hydroxylating dioxygenase subunit alpha	92	*Pseudomonas fluorescens* WP_031319057.1	Methyxanthine N1-demethylation
*orf11*	VOC family protein	83	*Pseudomonas fluorescens* WP_057008287.1	Metal dependent vicinal oxygen chelating enzyme
*orf12*	S-(hydroxymethyl) glutathione dehydrogenase	93	*Pseudomonas fluorescens* WP_046819641.1	Involved in formaldehyde metabolism

Note: Determination of homologous genes for each *orf* was conducted by performing a BLAST against the sequences available in the NCBI database.

**Table 3:** Kinetic parameters of the enzymatic reaction of TMU to methyluric acid [50].

Enzyme	Substrate	*K_m_* (μM)	*k_cat_* (min^−1^)	*k_cat_*/*K_m_* (min^−1^·μΜ^−1^)
TmuM-His_6_	Trimethyluric	10.2 ± 2.2	448.9 ± 21.7	44.1 ± 2.1
	1,3-dimethyluric acid	126.5 ± 29.3	185.0 ± 16.4	1.5 ± 0.1
	3,7-dimethyluric acid	1.3 ± 0.6	118.2 ± 8.4	89.4 ± 6.3
	1-methyluric acid	1.2 ± 0.5	29.1 ± 2.4	24.5 ± 2.0
	Uric acid	NA	–	NA

**Table 4:** Functional prediction of genes involved in the C-8 pathway in the genome of *Pseudomonas* sp. CBB1 [50].

Gene	Homologous Protein	% Identity	NCBI Accession Number	Proposed Function
*tmuH*	Hydroxyisourate hydrolase	50	YP_002976942	TM-HIU hydrolase
*tmuD*	OHCU decarboxylase	39	YP_003695277	TM-OHCU decarboxylase
*tmuM*	FAD-binding monooxygenase	38	YP_003741647	TMU monooxygenase
*cdhA*	Xanthine dehydrogenase Molybdopterin-binding protein	49	YP_003395893	Cdh molybdopterin-binding subunit
*cdhB*	Alcohol dehydrogenase medium subunit	39	ADV16272	Cdh FAD-binding subunit
*cdhC*	Aldehyde oxidase small subunit	59	YP_002521823	Cdh [2FE-2S]-binding subunit

## Data Availability

No new data were created or analyzed in this study. Data sharing is not applicable to this article.
